# RETRACTION: Efficacy of Faecal Microbiota Transplantation for the Treatment of Autism in Children: Meta‐Analysis of Randomised Controlled Trials

**DOI:** 10.1155/ecam/9826719

**Published:** 2026-07-16

**Authors:** Evidence-Based Complementary and Alternative Medicine

RETRACTION: D. Zhu, X. Jin, P. Guo, et al., “Efficacy of Faecal Microbiota Transplantation for the Treatment of Autism in Children: Meta‐Analysis of Randomised Controlled Trials,” *Evidence-Based Complementary and Alternative Medicine*, vol. 2023 (2023), https://doi.org/10.1155/2023/5993628.

The above article, published online on 9 February 2023 in Wiley Online Library (https://wileyonlinelibrary.com), has been retracted by John Wiley & Sons Ltd.

The retraction has been agreed following an investigation undertaken by the publisher, which identified concerns related to the scientific content of the article. More specifically, the meta‐analysis included 3 papers, which did not meet the primary inclusion criterion of being randomized controlled trials.

As a result of the investigation, the conclusions of this article are considered unreliable and the article is therefore retracted.

The authors were contacted to elaborate on the above concerns and agreed with the retraction of the article.

